# Role of grain size on the corrosion resistance of pipeline steels in acidic environment

**DOI:** 10.1186/s40712-025-00240-9

**Published:** 2025-02-21

**Authors:** Meekness Nnoka, Tonye Alaso Jack, Sandeep Yadav, Jerzy Szpunar

**Affiliations:** Department of Mechanical Engineering, University of Saskatchewan, 57 Campus Drive, Saskatoon, SK S7N 5A9 Canada

**Keywords:** Pipeline steel, Microstructure, Grain size, Corrosion, Passivation, Acidic environment

## Abstract

The microstructure of pipeline steels plays an important role in determining their resistance to corrosion. Among various microstructural features, grain size has been a topic of ongoing debate regarding its influence on the corrosion resistance of pipeline steels. While it is established that grain size inversely correlates with yield strength, its impact on corrosion resistance in acidic environments remains unclear. This study investigated the effects of grain size on the corrosion resistance of pipeline steels exposed to an acidic environment. Heat treatments were conducted to vary the grain size from 8 to 19 µm while minimizing contributions from other microstructural features. The findings revealed that reducing grain size significantly enhanced corrosion resistance by promoting passivation. Grain boundaries were identified as the preferred sites for forming protective oxide layers, compared to grain interiors. Consequently, samples with finer grains, which inherently possess a higher density of grain boundaries, exhibited enhanced passivation, resulting in greater surface coverage by protective oxide layers. In contrast, samples with larger grains primarily formed oxide layers along the grain boundaries, leaving the grain interiors more susceptible to attack by corrosive species. Additionally, a phenomenological model was developed based on the experimental results. This model was validated through independent measurements, confirming that passivation coverage increases with decreasing grain size in acidic environment.

## Introduction

Microstructure plays a pivotal role in the corrosion and cracking resistance of pipeline steels, dictating the vulnerability to environmentally induced degradation mechanisms. Microstructure encompasses a range of parameters such as phase composition, grain size and grain boundary characteristics, dislocation density, and crystallographic texture, all of which have been reported to impact the corrosion and cracking resistance of pipelines (Ohaeri et al. 2018a; Sofronis and Robertson 2006; S Lynch 2003; Ghosh et al. 2018; Kadhim and Dr.M.T. Ali 2021; Nnoka et al. 2024). Optimizing the microstructural parameters obtained after thermomechanical processing and microalloying holds the potential to significantly improve the corrosion resistance of pipeline steels.

For instance, some researchers suggested that the < 110 >||ND and < 111 >||ND textures are less prone to corrosion (Ohaeri et al. 2020; Wang et al. 2021). In terms of phase composition, martensitic constituent shows poor corrosion resistance when compared to other secondary phases (Katiyar et al. 2019). Few researchers also hinted that low dislocation density favors corrosion resistance (Peng-Xian 2013; Rodionova et al. 2020).

However, despite the influences of several microstructural features on corrosion, the role of grain size on corrosion susceptibility seems unclear and highly debated.

There is also scarce literature on the role of grain size in the passivation and corrosion susceptibility of pipeline steels. From a few studies, it is generally believed that the corrosion rate increases as grain size increases (Rodionova et al. 2020; Soleimani et al. 2020; Ohaeri et al. 2018b; Eliyan et al. 2017). That is, the corrosion resistance of a material is directly proportional to its grain size. However, corrosion remains a multifaceted subject with pipeline steels showing different corrosion behavior in different corrosion environments (Yadav 2023; Jack and Szpunar 2025). This suggests a more detailed investigation relating the corrosion environment to the corrosion susceptibility of pipeline steels.

Maryam et al. studied the effects of grain size on pipeline steel corrosion resistance in a 3.5 wt% NaCl solution at room temperature (Soleimani et al. 2020). The authors investigated the corrosion resistance of pipeline steel samples of various grain sizes. They concluded that two distinct stages for the dependency of corrosion current density (*i*_corr_) on grain size exist. The authors revealed that above a limiting average grain size of ∼22 μm, *i*_corr_ decreased slowly with increasing grain size. Below this limiting value, *i*_corr_ increased rapidly, which was related to the increased density of grain boundaries as interpreted by theoretical calculation of the number of grains per unit area. While the issue of limiting average grain size remains controversial, the authors did not provide a reason for such a shift. The absence of crystallographic texture and internal strain analysis of the samples produced for their experiments raises questions on the contribution of other microstructural features to their conclusions, as these can also impact the corrosion resistance of pipeline steel (Wang et al. 2021; Ohaeri et al. 2018b).

Eliyan et al. (Eliyan et al. 2017) demonstrated passivation in API-X100 steel using cyclic voltammetry in aerated bicarbonate-carbonate solutions. They found that higher carbonate concentrations slowed dissolution and enhanced passive film formation, while lower bicarbonate concentrations disrupted it. However, at higher bicarbonate levels, passivation improved, raising the transpassive potential. This suggests that passivation occurs in both acidic and basic environments, with more pronounced effects in acidic conditions according to the literature (Ohaeri et al. 2018b; Yadav 2023; Jack and Szpunar 2025; Zhang et al. 2009; Torres-Islas et al. 2008).

Mishra et al. (Mishra et al. 2020) also confirmed the effect of passivation by utilizing electrochemical modeling of electrochemical impedance spectroscopy (EIS) to show that the metal dissolution rate is limited by slow inward diffusion of carbonate ions (CO_3_^−2^) through a porous precipitated corrosion product layer to the API X70 pipeline steel surface during active dissolution in 1 M NaHCO_3._

In another study, Wang et al. investigated the corrosion properties of fine and coarse-grained pipeline steel samples (Wang et al. 2021). According to their research, grain refinement decreased corrosion resistance in the NaCl solution but improved corrosion resistance in the NaHCO_3_ solution. According to the authors, coarse-grained pipeline steel is known to have anodic passivation, while refined grain pipeline steel can induce self-passivation.

Interestingly, this was also in line with Jack and Szpunar’s report on the corrosion resistance of similar steels having ununiform grain sizes (Jack and Szpunar 2025). The authors noted a significant level of passivation in the area characterized by a higher density of grain boundaries, contrasting with minimal passivation in regions with larger grain sizes and fewer grain boundaries. However, they noted that unlike in the NaHCO_3_ solution, no hydrogen evolution was observed during the corrosion process in the NaCl corrosion medium. Consequently, this led to reduced passivation and heightened corrosion susceptibility. Hence, the emission of hydrogen during the corrosion process facilitates greater passivation, thereby enhancing corrosion resistance. Interestingly, this passivation phenomenon is predominant in acidic media.

It is generally believed that improved corrosion resistance comes from having more grain boundaries, which helps surfaces passivate more easily (Kus et al. 2006; Miyamoto 2016; Tsai and Chuang 1997). However, these studies rarely correlate how these responses are connected to grain size, passivation, and the specific corrosion environment.

Ralston et al. (Ralston et al. 2010) proposed a phenomenological model showing the relationship between corrosion current density (*i*_corr_) and grain size, presented in Eq. (1). The authors obtained this relationship by collating data from several works in the literature with *i*_corr_ ≤ 10 µA cm^−2^.

1 $$i_{corr=\;(A)\;+\;(B)\;{\;grain\;size}^{-0.5}}$$

where “*A*” is a constant representing the contributions of the corrosive media and “*B*” accounts for variations in material composition or impurity level.

Although the correlation between grain size and *i*_corr_ presented by Ralston et al. (Ralston et al. 2010) seems convincing, it spans a wide variety of corrosive environments, from acidic to alkaline solutions. This suggests that the range of the constant A, which accounts for the contributions of the corrosive medium, could vary significantly. Considering the subtle differences in how electrolytes with varying pH levels interact with steels, it is noted that passivation tends to occur more readily in hydrogen-producing electrolytes (acidic solutions), and this effect diminishes with a decrease in pH or hydrogen production during corrosion (Ohaeri et al. 2018b; Yadav 2023; Jack and Szpunar 2025; Yavas et al. 2018; Ralston and Birbilis 2010). Therefore, it is essential to treat these environments individually to deepen our understanding of steel behavior within them. As the microstructure of the steel changes, the interaction and response of the steel can become increasingly complex, indicating that the constant B in the Ralston equation could also exhibit significant variability.

Given the ongoing debates regarding the influence of grain size on corrosion, this research seeks to provide insights into this topic, focusing more specifically on oil exploration environments, which are typically acidic and where steel passivation is anticipated.

With the lack of a clear consensus on the effect of grain size on corrosion especially in acidic environment, it becomes crucial to investigate and clarify its role in the corrosion and passivation process. This entails altering the grain size of the steel while minimizing changes to other critical microstructural aspects such as dislocation density, phase composition, and crystallographic texture. Additionally, it requires exposing the steel to a specific acidic solution, considering that different electrolytes would possess distinct corrosion and passivation characteristics. Thus, this will contribute to the development of a phenomenological model to describe the relationship between grain size and passivation under these conditions. To the best of our knowledge, no such relationship has been reported previously.

Hence, the focus of this study is to investigate the effect of grain size on the corrosion resistance of pipeline steels in acidic environments, with particular attention to its relationship with passivation. It also focuses on proposing a phenomenological model to define the relationship between grain size and passivation in acidic environment.

## Materials and methods

### Steel samples

The chemical composition of the X65 pipeline steel plate used for this study is given in Table 1.

**Table 1:** Chemical composition of the X65 pipeline steel (wt%)

C	Si	Mn	P	S	Nb	Ti	Cr	Ni	Cu	Fe
0.05	0.20	1.15	0.009	0.0012	0.019	0.016	0.18	0.29	0.19	Bal

The hot-rolled plate was cut into long strips with 4 mm thickness. These strips were obtained from the surface of the steel plate. These strips were annealed at different temperatures above the recrystallization temperature in a bid to vary the grain size of the samples. The microstructural characteristics of the annealed steel samples were examined using optical microscopy and electron backscatter diffraction (EBSD). The topmost layer of the annealed samples was used for the electrochemical corrosion test. To examine the effect of grain size on corrosion, a surface phenomenon, thermodynamic simulations were conducted using JMatpro. The simulations identified temperatures above the Ar3 transformation point to allow for variations in grain size while minimizing alterations to other microstructural features. Additionally, the Arrhenius relationship between grain boundary migration and temperature influenced the selection of annealing temperatures, as higher temperatures enhanced grain boundary diffusion, leading to an increase in grain size.

**Fig. 1:**
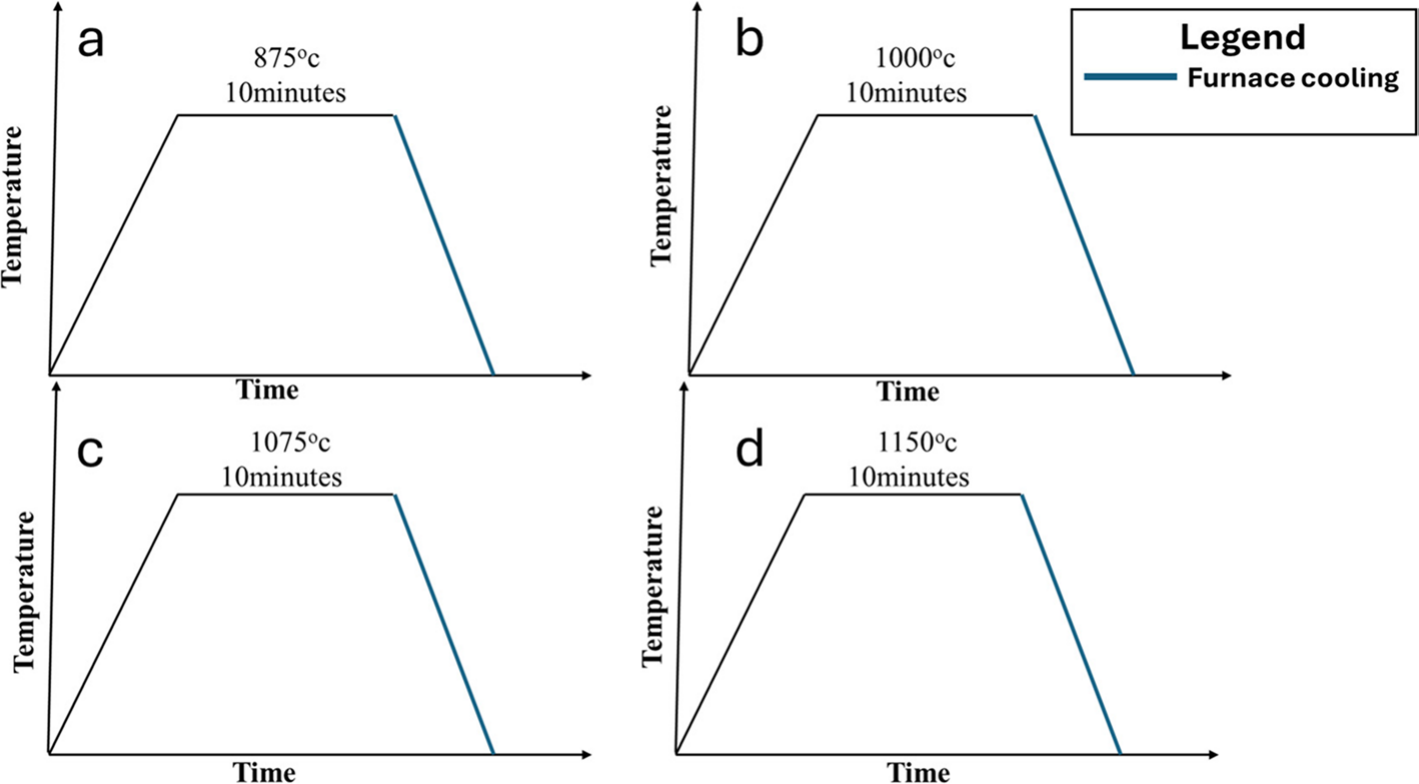
Schematic illustration of the full annealing heat treatment schedules applied to the steel samples: **a** 875s1, **b** 1000s1, **c** 1075s1, **d** 1150s1

The schedules for the full annealing treatment of the steel samples for this study are illustrated in Fig. 1 and are described as follows:

The steel samples were heated up in a pre-heated furnace at four different temperatures, 875 °C, 1000 °C, 1075 °C, and 1150 °C, and held at these temperatures for 10 min. Upon reaching the set holding time, the heating process was followed by furnace cooling. The resulting annealed samples are designated as 875s1, 1000s1, 1075s1, and 1150s1, respectively, in this work.

### Microstructural evaluation

The microstructural analysis for all steel samples was performed on the rolling direction–transverse direction (RD-TD) plane. Proper metallographic procedures were followed during the preparation of the steel samples for microstructural characterization. The samples were progressively polished using 180, 320, 400, 600, 800, 1200, 2400, and 4000 grit SiC papers, followed by 3 µm MD-Dac and 1 µm MD-Nap diamond polishing discs to achieve a smooth surface. The samples for optical microscopy were etched using Nital solution, and an Olympus BX-KMA-LED light microscope was used to image their microstructures. In addition, SEM images were also captured using Hitachi SU6600 scanning electron microscope.

The samples for EBSD were further vibratory polished after diamond polishing in a 0.04 µm colloidal silica slurry. A Hitachi SU 6600 field emission SEM with an Oxford Instrument Nordlys nano EBSD detector was used. A pre-tilted sample holder placed the sample at an inclination of 70° from the horizontal plane during the EBSD measurements. The diffraction patterns were acquired and indexed using the AZTEC data acquisition software. The EBSD data collected was post-processed to obtain information on the crystallographic texture, grain size, and internal strain of the steel samples.

### Electrochemical corrosion studies

Electrochemical impedance spectroscopy (EIS) and potentiodynamic polarization were performed on the steel samples using a Gamry Instrument’s Interface 1010E Potentiostat and a three-electrode setup. The three-electrode setup comprised of the steel sample as the working electrode, a saturated calomel electrode (SCE) as the reference electrode, and a graphite rod as the counter electrode. Each specimen was ultrasonically degreased in acetone solution for 30 min before the commencement of the electrochemical test. The sample dimensions for the corrosion test were 20 mm × 20 mm × 2 mm, but only a 1 cm^2^ area on the polished surface was exposed to the electrolyte, using an electrochemical mask. An acidic electrolyte was used, comprising of 5 wt% of sodium chloride (NaCl), 0.5 wt% of CH_3_COOH, 1 wt% of H_2_SO_4_, and 3 g/l of ammonium thiocyanate (NH_4_SCN). This resulted in a pH of 1.3 for the electrolyte, which was used to simulate a sour-corrosive environment, as done previously in the literature (Ohaeri et al. 2018b; Jack and Szpunar 2025).

The open circuit potential (OCP) was allowed to stabilize for a period of 2 h before the commencement of the electrochemical corrosion tests. The EIS data acquisition was performed by applying a sinusoidal AC excitation of 10 mV (rms) amplitude, with the frequency ranging from 10 kHz to 10 mHz at 10 points per decade, with reference to OCP. For the potentiodynamic polarization tests, the samples were polarized within a potential range of ± 250 mV at a scan rate of 0.5 mV/s. The corrosion products after the polarization tests were also analyzed using Rahman Spectroscopy. Also, the corrosion rate of all annealed specimens was obtained using Eq. (2) (S.T. 2000).

2 $$\mathrm{CR}\;=\;\frac{0.14567\;\times\;{\mathrm i}_{\mathrm{corr}}\;\times\;eq.\mathrm W}D\;\mathrm{in}\;\mathrm{mpy}\;(\mathrm{mils}\;\mathrm{per}\;\mathrm{year})$$

where, *i*_corr_ = corrosion current density [μA/ cm^2^], eq.W = equivalent weight of corroding species [g], and *D* = density of steel sample [g/cm^3^].

## Results and discussions

### Microstructure of the as-received sample

**Fig. 2:**
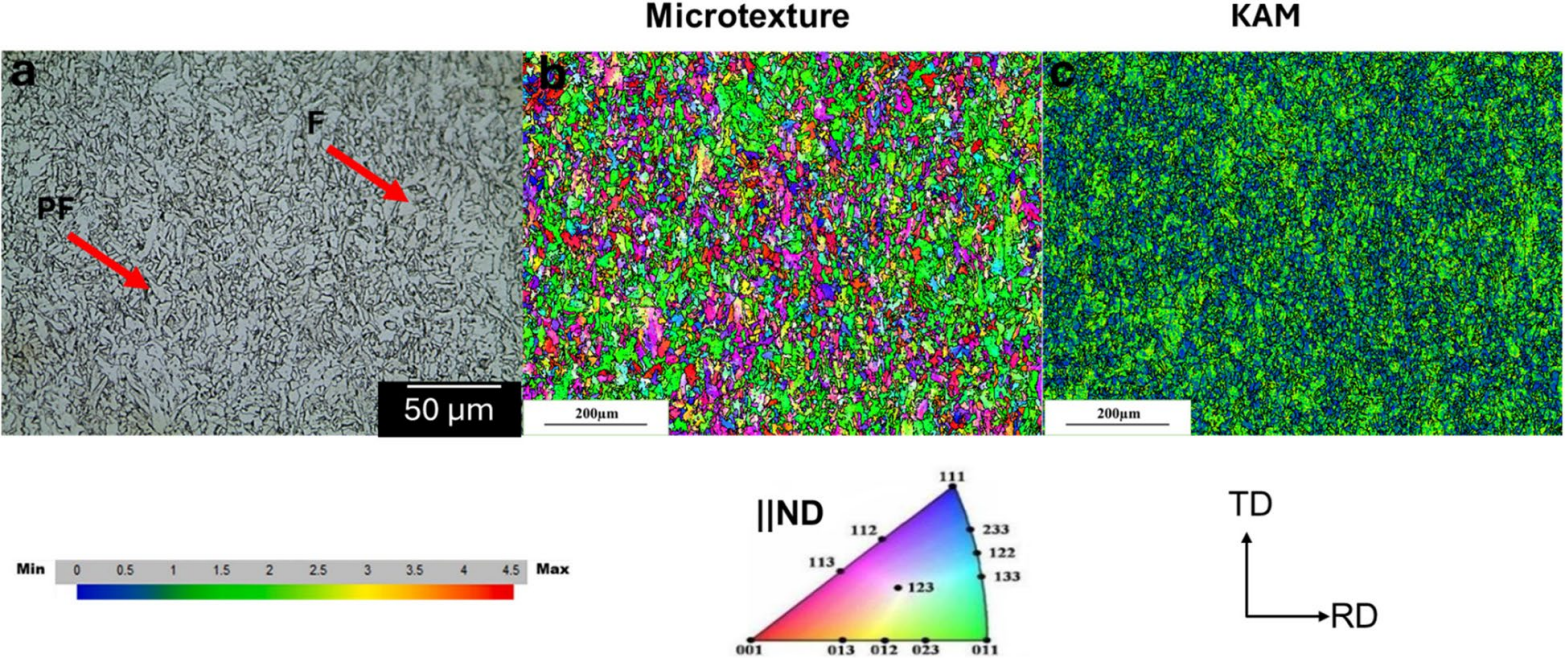
Microstructural parameters of the as-received sample: **a** optical micrograph, **b** micro texture, and **c** Kernel average misorientation (KAM)

Prior to the annealing treatments, microstructural characterization was conducted on the hot-rolled steel samples. Figure 2 shows the optical micrograph and EBSD maps showing the inverse pole figure parallel to ND (micro-texture), and the local misorientation for the surface layer of the as-received sample.

From Fig. 2a, it is evident that the surface layer of the as-received sample has a large fraction of < 110 >|| ND-oriented grains (green color). Figure 2b reveals that the surface layer of the as-received sample has a high KAM value, suggesting high dislocation density, whereas the optical micrograph as shown in Fig. 2c indicates the coexistence of ferrite (F) and some pearlite (P) in the structure of the as-received sample. Finally, the grain size of the sample was measured using the mean linear square method. The measured grain size was 3.4 ± 0.3 µm.

### Microstructure of annealed samples

**Fig. 3:**
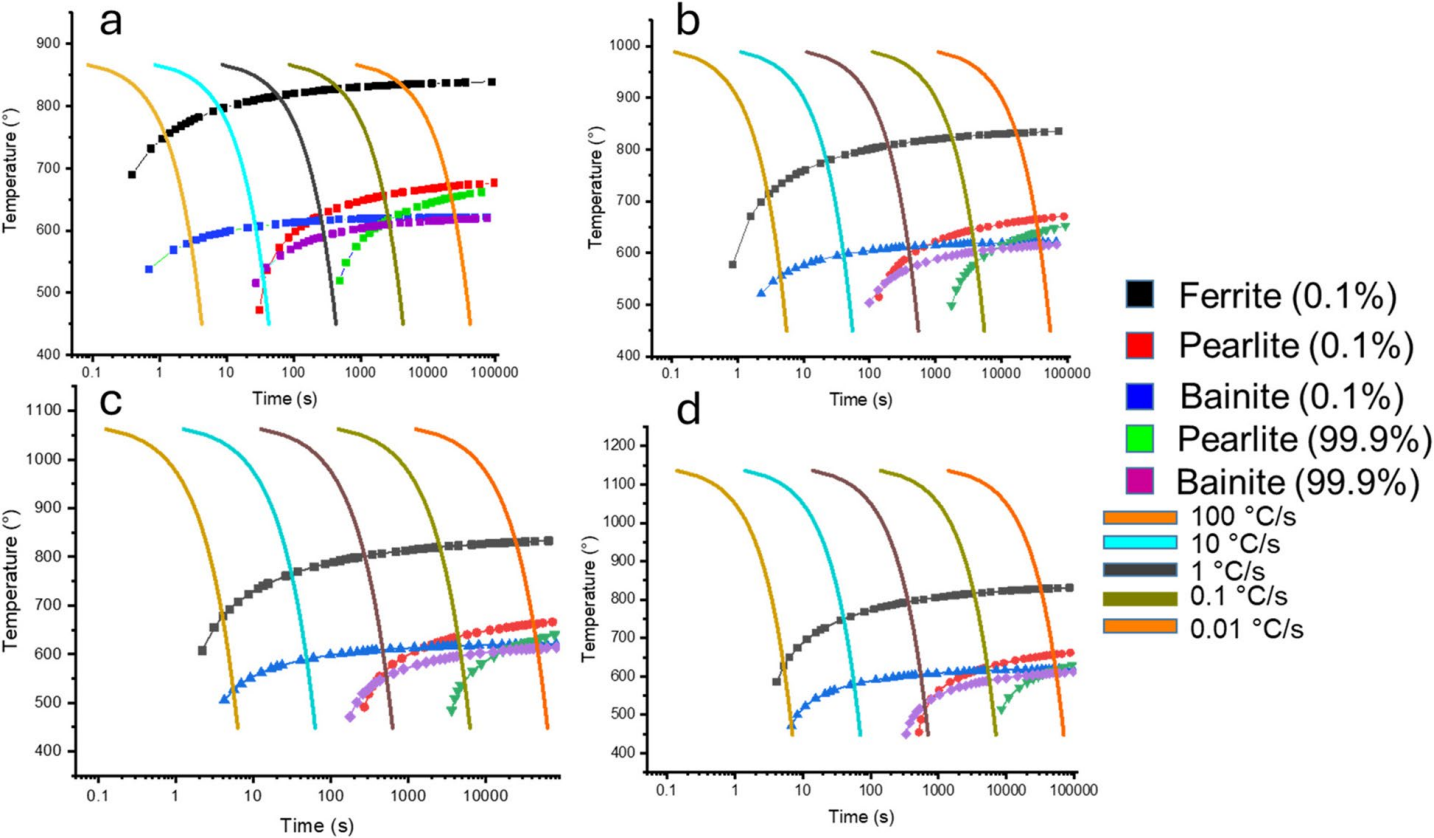
Simulated CCT diagrams for all annealed samples using JMatPro simulation software: **a** 875 °C, **b** 1000 °C, **c** 1075 °C, and **d** 1150 °C

To predict the microstructural phase composition based on the different annealing temperatures chosen for the steel strips, the JMatpro software was used to construct the continuous cooling transformation (CCT) curve for each heating condition, as shown in Fig. 3. Furnace cooling is known to occur at a very slow rate, some researchers (Dong et al. 2021; Jia-li et al. 2013) have quoted this rate to be about 0.03 C/s for steel samples which can also go as low as 0.01 C/s. Following the CCT diagrams generated by the JMatPro process simulation with a cooling rate of 0.01 C/s, it is predicted that all the steel annealed samples irrespective of their annealing temperature would primarily have a structure consisting of mainly polygonal ferrite and some pearlite. However, experimental microstructural analysis was also conducted on the annealed samples to validate these predicted microstructures.

**Fig. 4:**
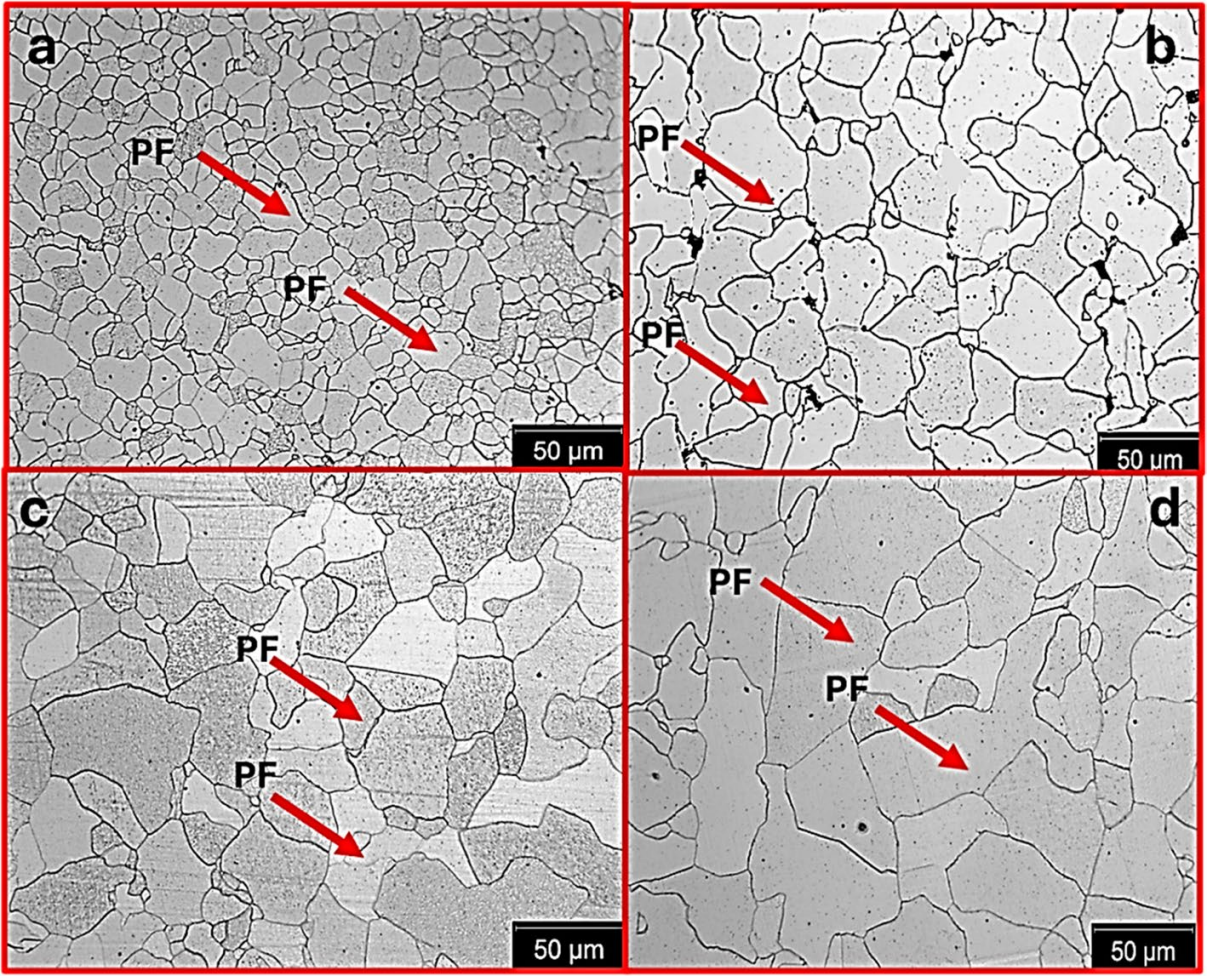
Optical microstructure of the annealed samples RD-TD plane, **a** 875 °C, **b** 1000 °C, **c** 1075 °C, **d** 1150 °C

Figure 4 shows the optical micrograph on the RD-TD plane of the annealed samples. The primary microstructures of the annealed plates on the RD-TD planes are polygonal ferrite. More importantly, the annealing treatments were successful in obtaining incremental grain sizes with increasing temperature. This phenomenon is because of grain boundary migration at higher temperatures, due to an increase in the kinetic energy of atoms, allowing atoms to move more readily across grain boundaries. As a result, atoms can more easily jump from one grain to another, enabling the migration of grain boundaries (Toriumi 1982). However, the rate and effectiveness of migration are influenced by factors such as temperature, applied stress, and the grain boundary characteristics. Other characterization techniques were conducted to ensure that the grain size of the annealed samples was the most varied microstructural feature.

## Estimation of the grain size of the annealed samples

The grain size of the annealed samples was determined using the mean linear square method (Designation: E112–12 standardtest methods for determining average grain size 1, (n.d.). xxxx), as well as from the EBSD data. The average grain sizes of the annealed samples using both methods were quite consistent and are presented in Table 2, along with their error margin. The largest grain size is 19 ± 1 µm corresponding to an annealing temperature of 1150 °C, and the lowest grain size is 8 ± 1 µm corresponding to an annealing temperature of 875 °C. The samples will be designated using their grain sizes: 8 µm, 11.5 µm, 13 µm, and 19 µm, respectively.

**Table 2:** Measured grain sizes of annealed samples

Annealing temperature (°C)	Measured grain size (µm)
875	8 ± 1
1000	11.5 ± 0.5
1075	13 ± 1
1150	19 ± 1

## EBSD analysis

**Fig. 5:**
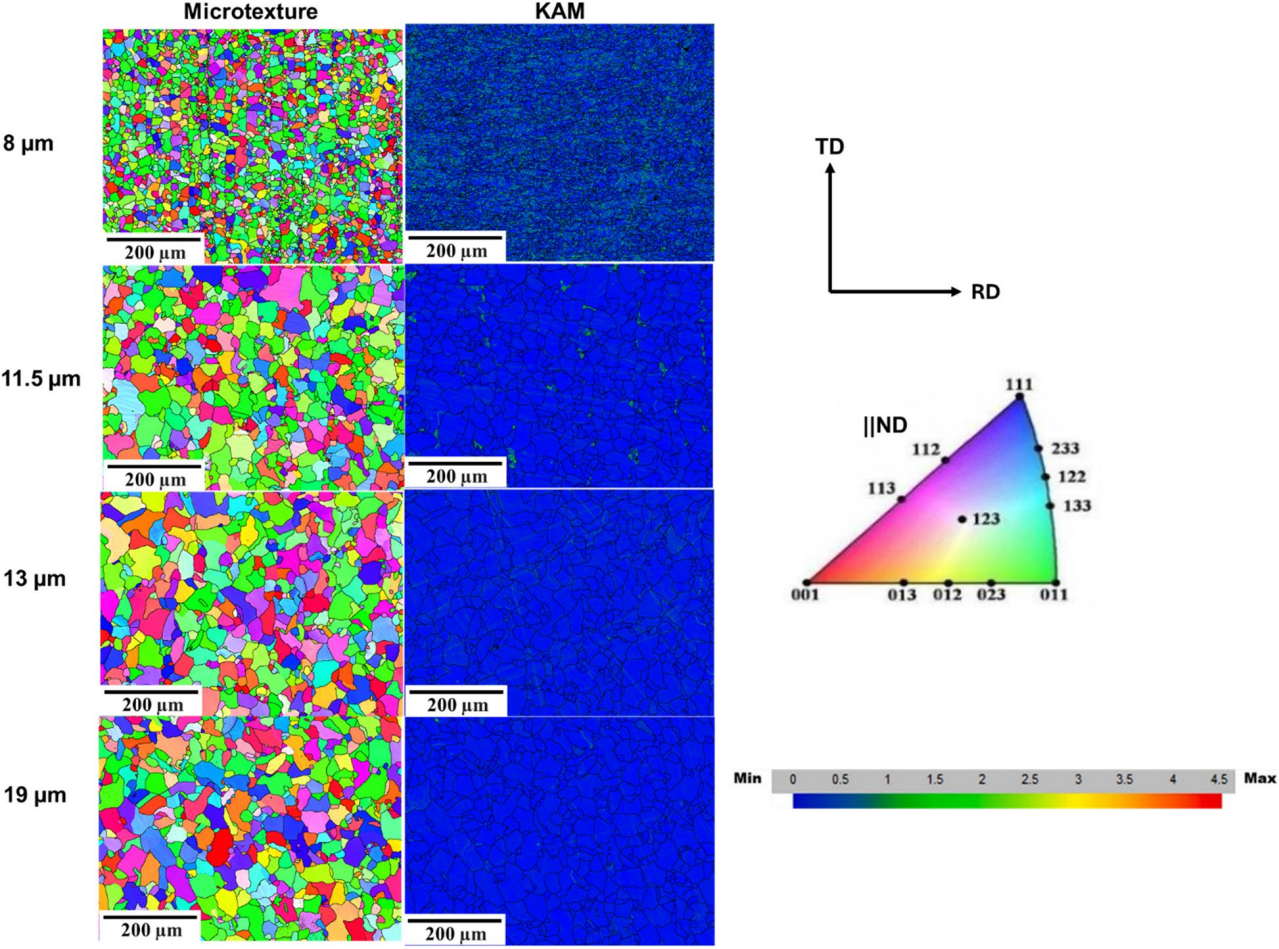
EBSD maps for all annealed samples on the TD-RD plane

Figure 5 shows the EBSD maps obtained for the annealed samples that represent the inverse pole figures parallel to ND (microtexture) and the KAM. From Fig. 5, it can be deduced that grain size differences observed in IPF maps are similar to the ones observed in optical micrographs of annealed samples. Individual grains having misorientation greater than 15° are separated by grain boundaries, which are shown as black lines.

From Fig. 5, it is evident that the surface layer of all samples has a large proportion of < 110 >|| ND-oriented grains (green color). This reveals that annealing did not significantly vary the crystallographic texture characteristics of the annealed samples. Few studies have shown that annealed pipeline samples usually inherit the texture character of the as-received material (Wang et al. 2020; Joodaki et al. 2017).

**Fig. 6:**
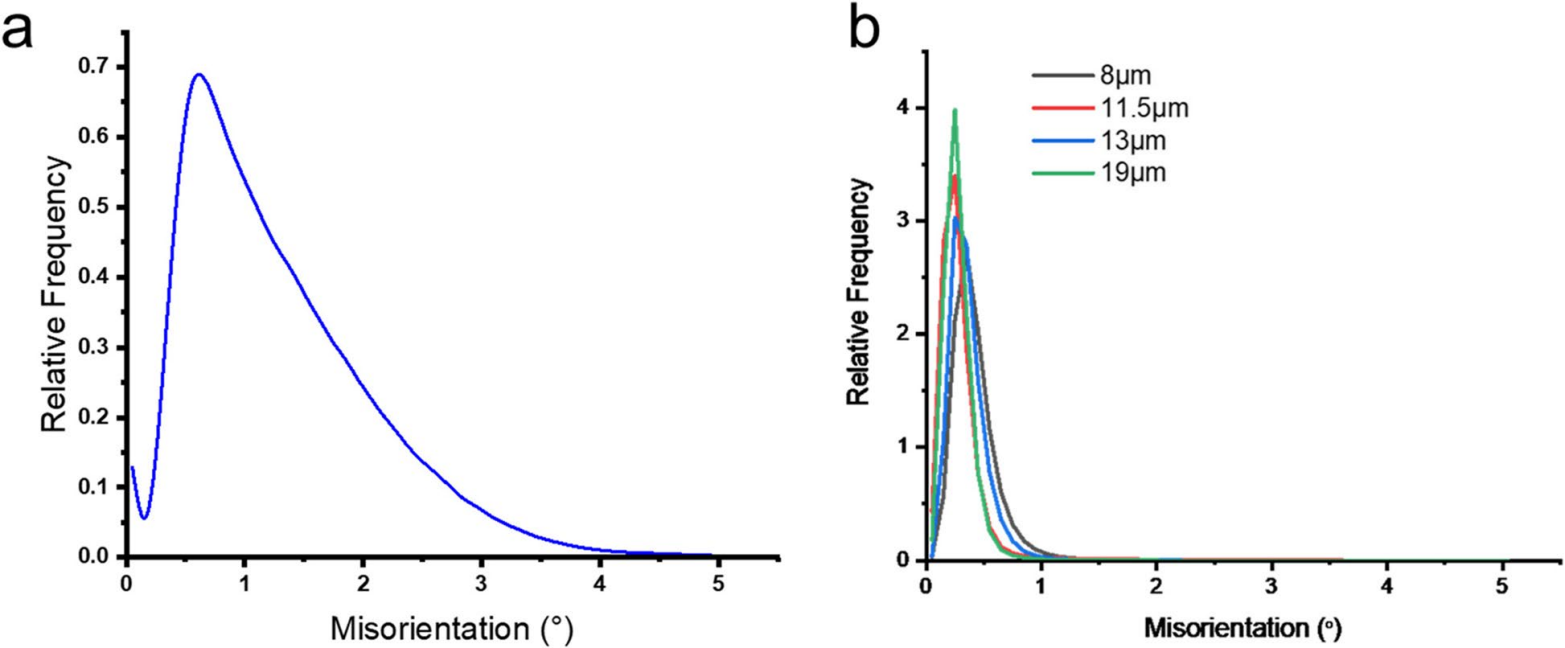
KAM distribution plots of **a** as-received sample and **b** annealed samples

Additionally, Fig. 6 shows the Kernel average misorientation (KAM) distribution plot for the as-received samples and the annealed samples. According to Fig. 6a, the surface (RD-TD plane) layers of the as-received specimen have a higher KAM value than that of all the annealed samples, as shown in Fig. 6b. This indicates that the average misorientation was significantly minimized due to the annealing effect. With the increase in annealing temperature, the grain size increased, and there was a significant reduction in average misorientation. Figure 6b also confirms that the reduction in dislocation density occurred in all the annealing conditions.

**Fig. 7:**
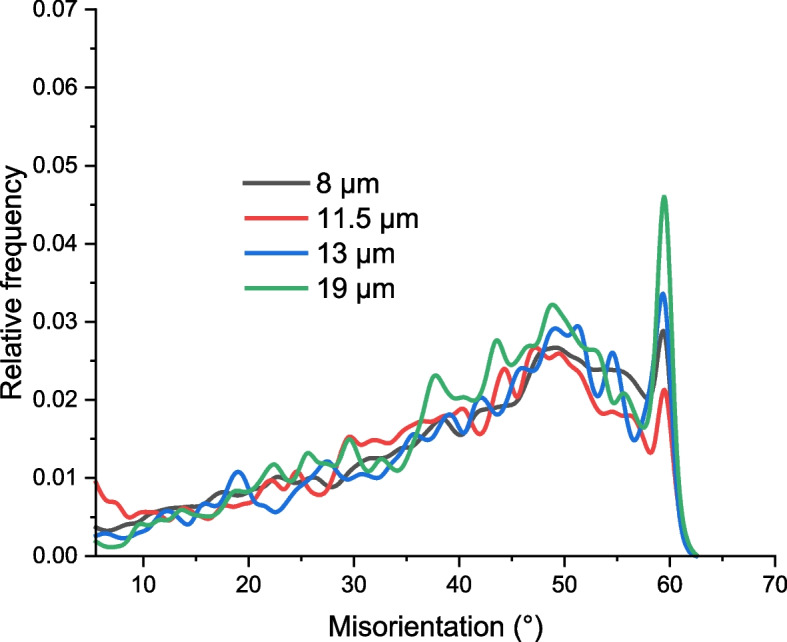
LAGB and HAGB plots for the surface layers of annealed samples

Overall, it can be deduced that dislocation density is low in all samples. Few studies (Chen et al. 1979; Momotani et al. 2020; Birnbaum 1994) have shown that high dislocation density favors corrosion, especially pitting corrosion (Miyamoto et al. 2019; Kayani et al. 2023). Therefore, it is necessary to optimize the cooling rate to optimize the dislocation density induced during TMCP. In this case, annealing significantly reduced dislocation density in the samples. Finally, Fig. 7 shows that the grain boundary character that reveals the low-angle grain boundary (LAGB) and high-angle grain boundary (HAGB) distribution of all annealed samples is very similar. Therefore, the most significantly changed microstructural parameter is the size of the grains.

### Electrochemical corrosion analysis

#### Open circuit potential

**Fig. 8:**
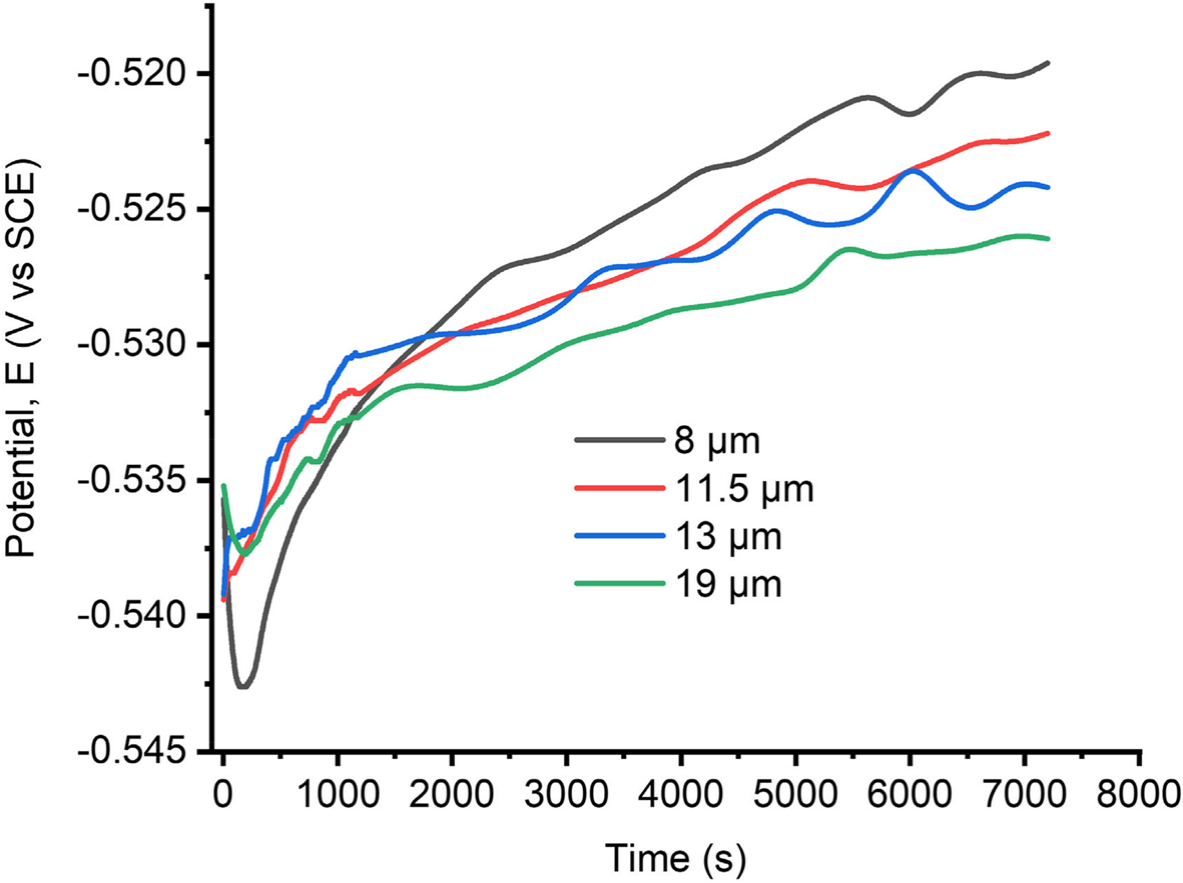
Open circuit potential curves obtained for the annealed samples in acidic media

The potential created between the pipeline samples and the corrosive medium is called the OCP. This potential is measured as the electrochemical reaction between the metal surface and the solution tends towards a state of equilibrium. OCP is measured when no external current or voltage is applied. Generally, OCP is a characteristic of the thermodynamic tendency of a sample to corrode. This is because more negative potentials usually indicate a higher likelihood of corrosion of the sample. According to several corrosion research, pipeline steel specimens with higher negative OCP have worse corrosion resistance, and vice versa (Ohaeri et al. 2020, 2018b; Yadav 2023; Jack and Szpunar 2025). In this study, the surfaces of annealed samples were given enough time to stabilize in the corrosive medium. A period of about 2 h was allowed for the samples to stabilize. Figure 8 shows the plot of potential versus time for all samples under the sour corrosive medium.

From Fig. 8, it can be deduced that the OCP curves for all annealed samples behaved similarly towards the end, but the beginning is very different. This can be attributed to the initial attack from the electrolyte, where samples having more grain boundaries experienced a higher drop in potential, and it stabilizes over time due to possible passivation. The potentials decreased initially, reaching a minimum, and then began to increase. The extent of the dissimilarity based on the OCP curve behavior is based on the passivation potential of the samples. The passivation phenomenon implies that in the acidic solution, the initial rate of anodic dissolution was larger than the rate of metal oxide layer formation, but after a given amount of time, the rate of anodic dissolution reached its peak (the lowest point of the curve). As a result, the rate of metal oxide layer development overtook the rate of anodic dissolution, and the OCP curve began to increase until it reached a steady state, as shown in Fig. 8. As a result, the oxide film/corrosion product continues to build, safeguarding the steel surface. Similar behavior of OCP was observed in the literature (Ohaeri et al. 2018b; Yadav 2023).

However, when the OCP curves of the samples are compared, it is seen that samples with larger grains have a higher negative potential than samples with smaller grain sizes. This demonstrates that samples with bigger grain sizes are more electrochemically active in sour environments than those with smaller grain sizes. As a result, the OCP curves indicate that samples with larger grain sizes have a higher corrosion susceptibility than samples with smaller grain sizes. This is because samples with larger grain sizes have a lower passivation potential. It should be noted that this conclusion agrees with studies in the literature (Jack and Szpunar 2025; Liu and Wu 2007; Gadala and Alfantazi 2015; Li et al. 2004) where higher hydrogen evolution or more acidic solutions are said to promote protective oxide layer formation, and this oxide formation was more pronounced for samples with smaller grain sizes.

It is important to understand that the acidity or the sourness of an environment increases the reactivity of certain species, such as hydrogen ions, with the steel surface. This better passivation proficiency for samples with smaller grains can be a result of increased diffusion of metal ions to the surfaces where oxides are formed (grain boundaries). This means that samples having high grain boundary densities would have more passivation. On the other hand, samples with larger grain sizes have few grain boundaries. This reduced grain boundary densities create less room for oxide formation, accelerating the corrosion process. Interestingly, the sour/acidic medium contains H_2_SO_4_, a strong acid with a significant propensity to dissociate into H^+^ ions. When H₂SO₄ dissociates into H⁺ and SO₄^2^⁻, these corrosion species create an aggressive environment that accelerates iron dissolution and gas evolution (H₂), while promoting the formation of corrosion products.

### Electrochemical impedance spectroscopy

**Fig. 9:**
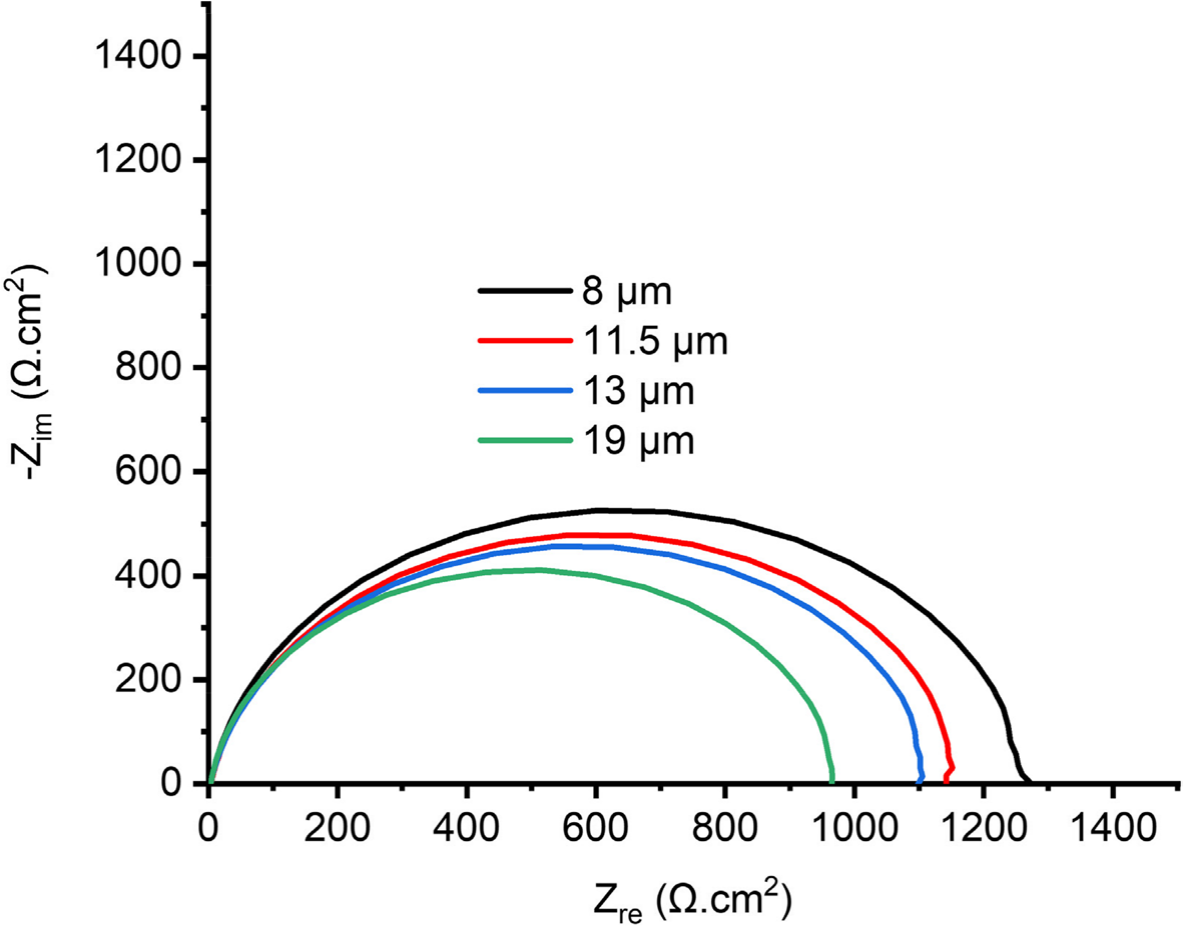
Nyquist curves obtained from the EIS test for samples in acidic media

Figure 9 shows the Nyquist curves obtained from the EIS tests done in the acidic media for all annealed samples. And from the Nyquist curves, the corrosion resistance of the samples was qualitatively determined.

It can be deduced from Fig. 9 that the diameter of the capacitive loop varies for all samples, while the charge transfer resistance of all annealed samples was high based on their different corrosion resistance. The EIS results show that samples with smaller grain sizes exhibited relatively higher corrosion resistance than samples with bigger grain sizes, which is in line with the OCP results.

### Potentiodynamic polarization (PDP)

**Fig. 10:**
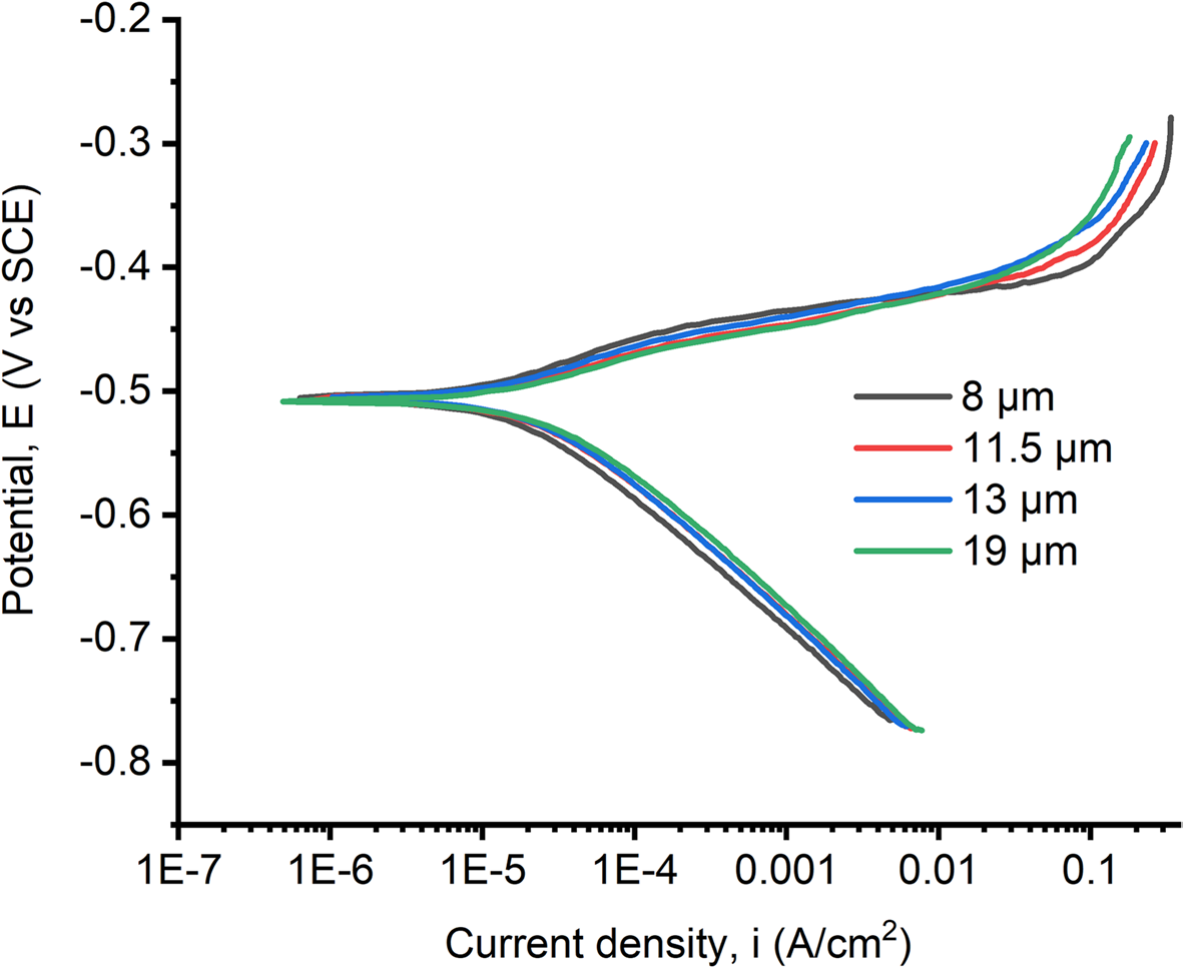
Potentiodynamic polarization curves for samples in sour/acidic media

Figure 10 shows the potentiodynamic polarization curves obtained to understand the corrosion behavior of the annealed samples in the acidic media with varying potentials applied. The applied potential gives rise to a current response that is measured as the voltage is swept continuously at a constant rate.

The electrochemical parameters were extracted from the potentiodynamic polarization curves using the Tafel extrapolation method, which includes the intersection of the linear parts of the Tafel curves, presented in Table 3. The corrosion rates of all the samples were calculated using Eq. 2. From Table 3, it is observed that the corrosion rate increased with increasing grain size.

**Table 3:** Electrochemical parameters of samples after potentiodynamic polarization

	***E***_**corr**_ **(mV)**	***i***_**corr**_ **(μA/ cm**^**2**^**)**	**Corrosion rate** **(mpy)**	***β***_**A**_** (mV/Decade)**	**β**_**C**_ **(mV/Decade)**
**8 µm**	− 497	10.2	4.65	48.30	85.66
**11.5 µm**	− 506	13.2	6.02	46.39	72.49
**13 µm**	− 507	17.2	7.85	63.84	95.65
**19 µm**	− 508	23.1	10.55	64.44	102. 86

**Fig. 11:**
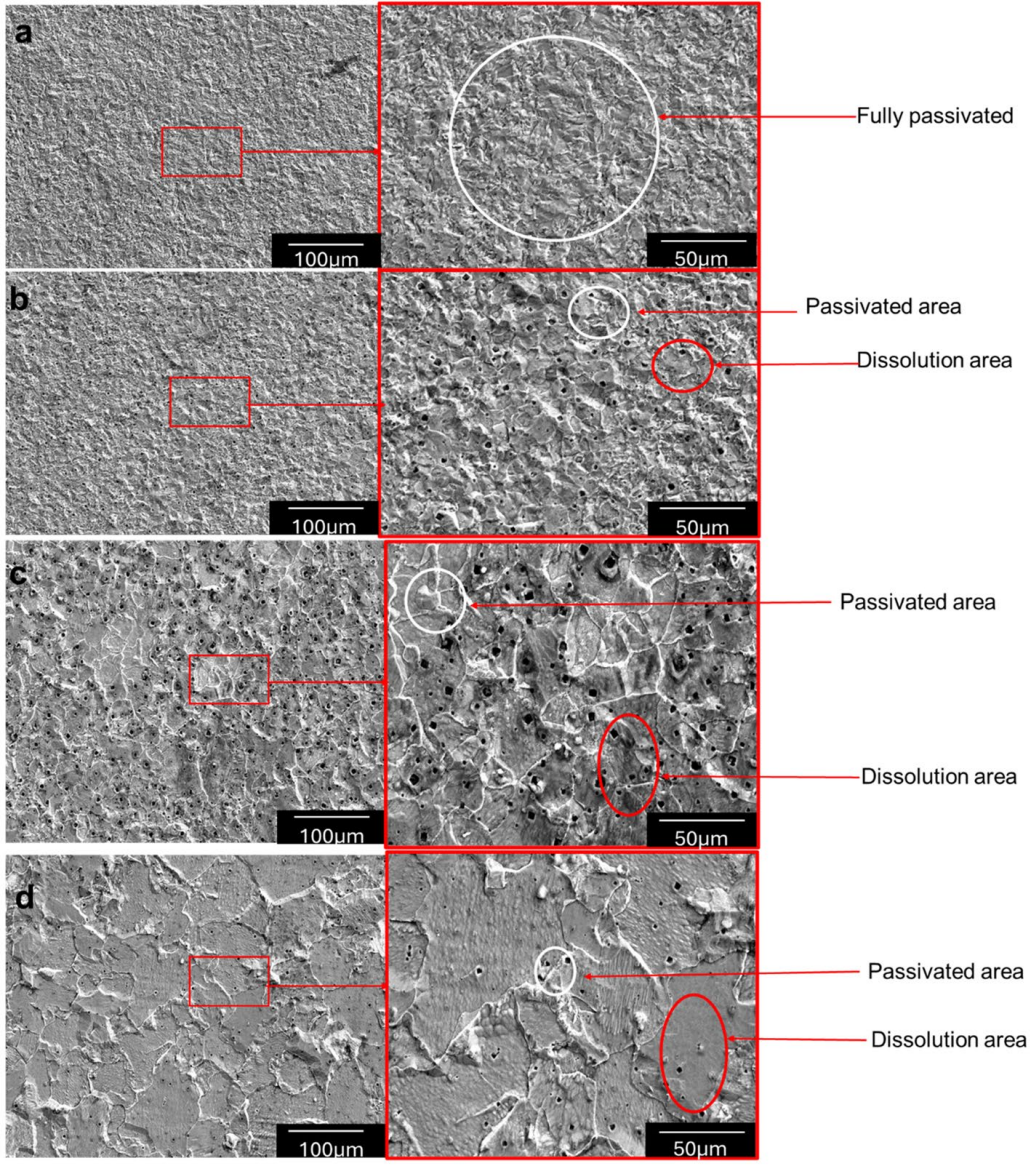
SEM micrographs of the corroded surfaces of the annealed samples after potentiodynamic polarization in acidic media **a** 8 µm, **b** 11.5 µm, **c** 13 µm, **d** 19 µm

Figure 11 shows the SEM images of all annealed samples after the electrochemical corrosion study. It can be seen from Fig. 11 that the samples having smaller grain sizes showed better resistance due to better passivation. This confirms that samples with smaller grains having more grain boundaries will have better passivation in acidic environment. This clear formation of protective layers on the grain boundaries of all samples can be visualized in Fig. 11.

**Fig. 12:**
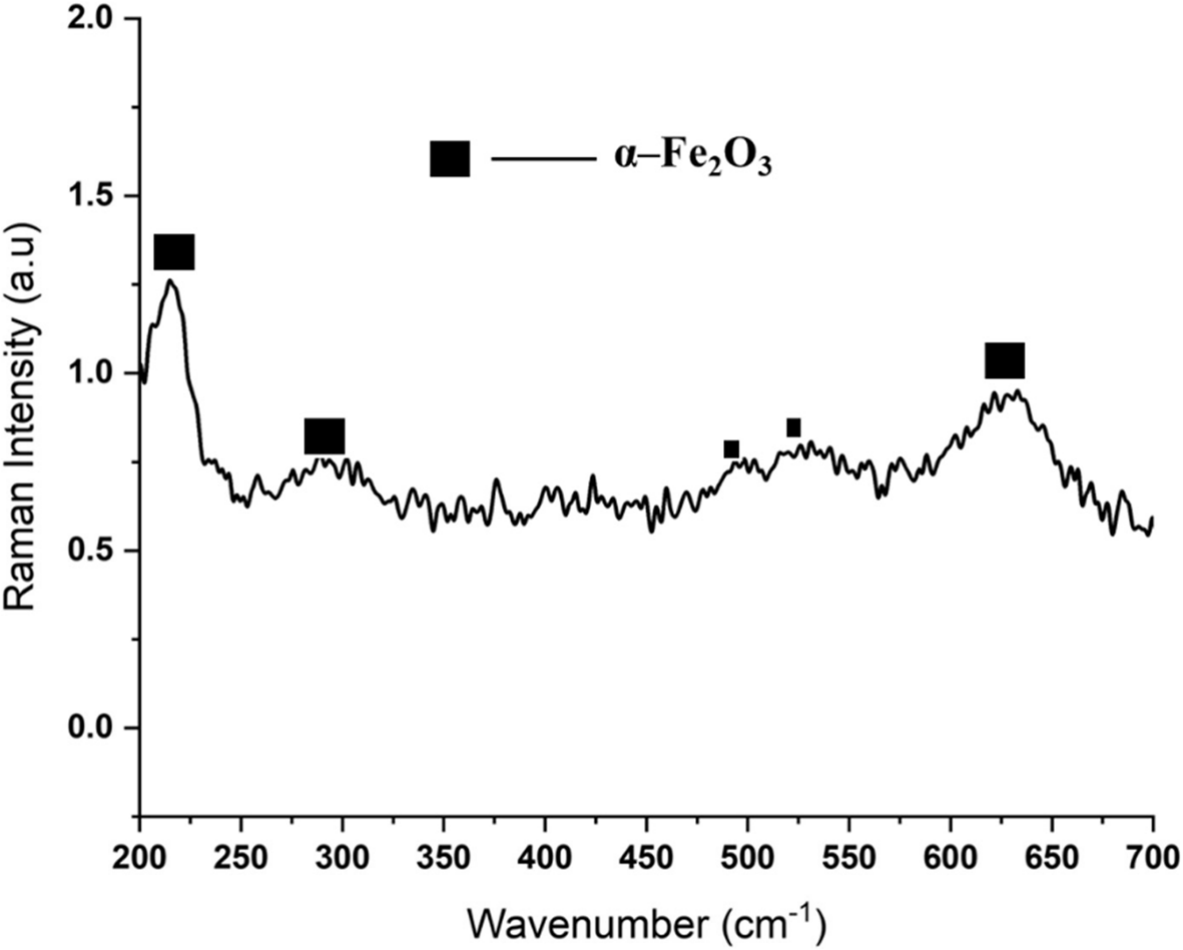
Representation of the Raman spectra of all annealed specimens

Figure 12 shows a representative of the Raman spectra of the annealed specimens after the electrochemical corrosion test. The passivation products were predominantly hematite (Fe_2_O_3_). The Raman spectrum shows the presence of five peaks around 220, 295, 494, 530, and 628 cm^−1^ which correspond to the Rahman shifts where Fe_2_O_3_ is formed (Hernández-Espejel et al. 2010; Heuer and Luttge 2018).

### Phenomenological modeling of the relationship between grain size, corrosion, and passivation

The experimental investigation conducted in this study is consistent with the findings of the literature, which demonstrate that steel samples with smaller grains have higher corrosion resistance than samples with larger grains in acidic environment. This better corrosion resistance has been clearly related to the increased density of grain boundaries acting as favorable sites for the formation of oxide films in this study, as shown in Fig. 11.

Understanding the relationship between grain size, corrosion, and passivation in acidic environment remains critical to this study. Thus, the passivation fraction should be accounted for to appreciate this relationship. Using image analysis software, the area fraction of the passivation layer for all annealed specimens was obtained and is presented in Table 4. The experimental results show that as the grain size decreases, the passivation fraction increases.

**Table 4:** Passivation fraction of annealed specimens

Grain size (µm)	Passivation fraction
8	0.89
11.5	0.53
13	0.42
16.6	0.29
19	0.23

The data clearly suggests an exponential relationship between passivation and grain size, as shown in Fig. 11 and Table 4, since the passivation fraction significantly drops from about 89% with a grain size of 8 µm to about 23% when the grain size increases to 19 µm.

This exponential relationship can be expressed with the power law approach, where the potential contribution from the grain interior is combined with the influence of the grain boundaries, resulting in the following equation:

3 $$P_{(d)}\;=P_\infty\;+\frac A{d^B}$$

where *P*_(d)_ is the passivation fraction as a function of grain size, *P*_∞_ is the passivation fraction or contribution for infinitely large grains, *A* is a constant representing the influence of grain boundaries on passivation, *d* is the average grain size, and *B* is an exponent that stabilizes the sensitivity of the passivation fraction to changes in grain size.

**Fig. 13:**
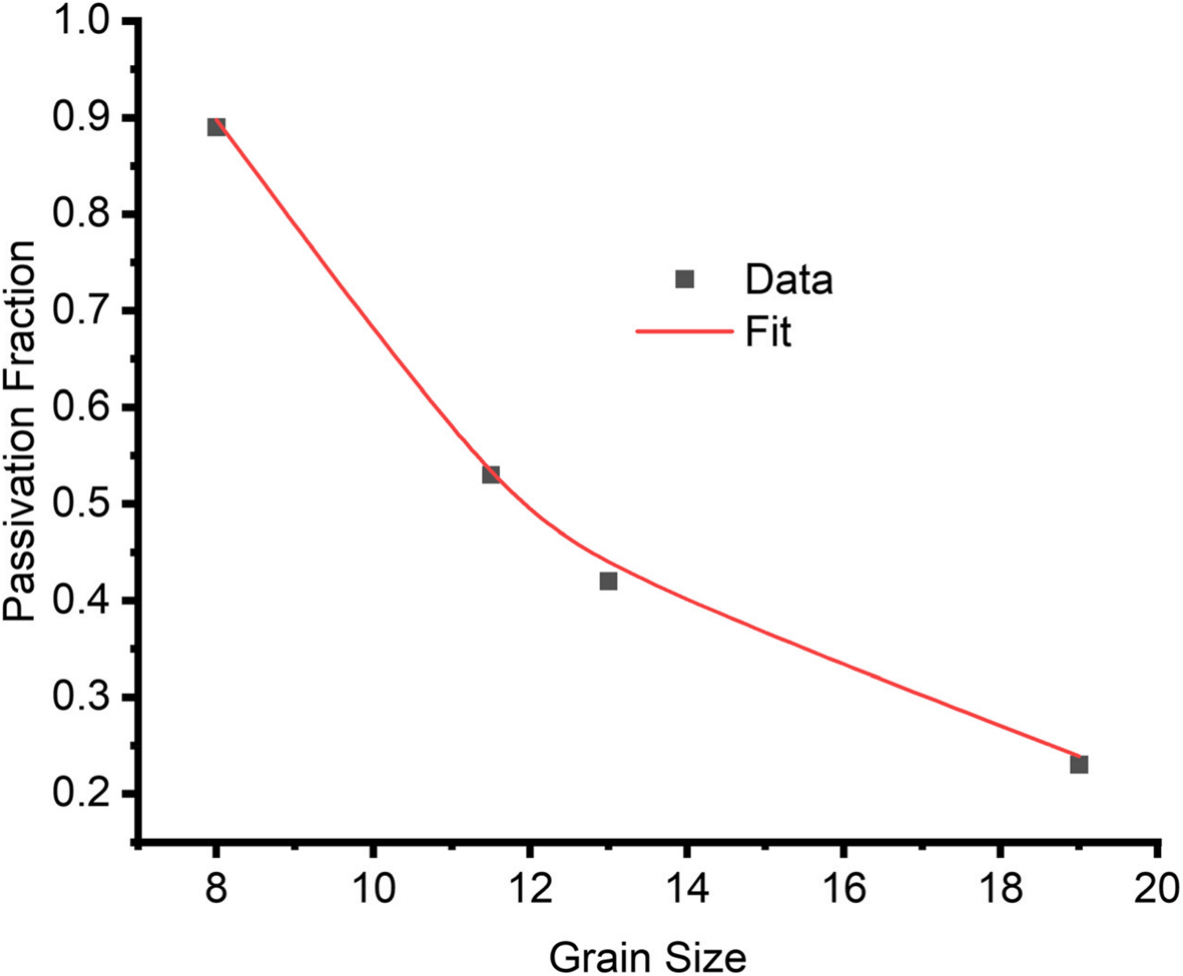
A plot of passivation fraction versus average grain size showing experimental and data fit for all annealed samples

A notable influence of the inverse of d accounts for the fact that as grain size reduces, the effect of grain boundary becomes significant. To confirm this relationship, the experimental data was fitted with the proposed phenomenological model, and it is presented in Fig. 13.

This model indicates that the influence of crystallographic orientation on passivation is limited, with larger grains having lower passivation fractions, implying that orientation effects are not as significant as that from the grain boundaries. Grain boundaries play an important part in the passivation process, with smaller grains exhibiting larger passivation fractions. This is because grain boundaries acting as active sites, which aid in the production of passivation layers.

**Fig. 14:**
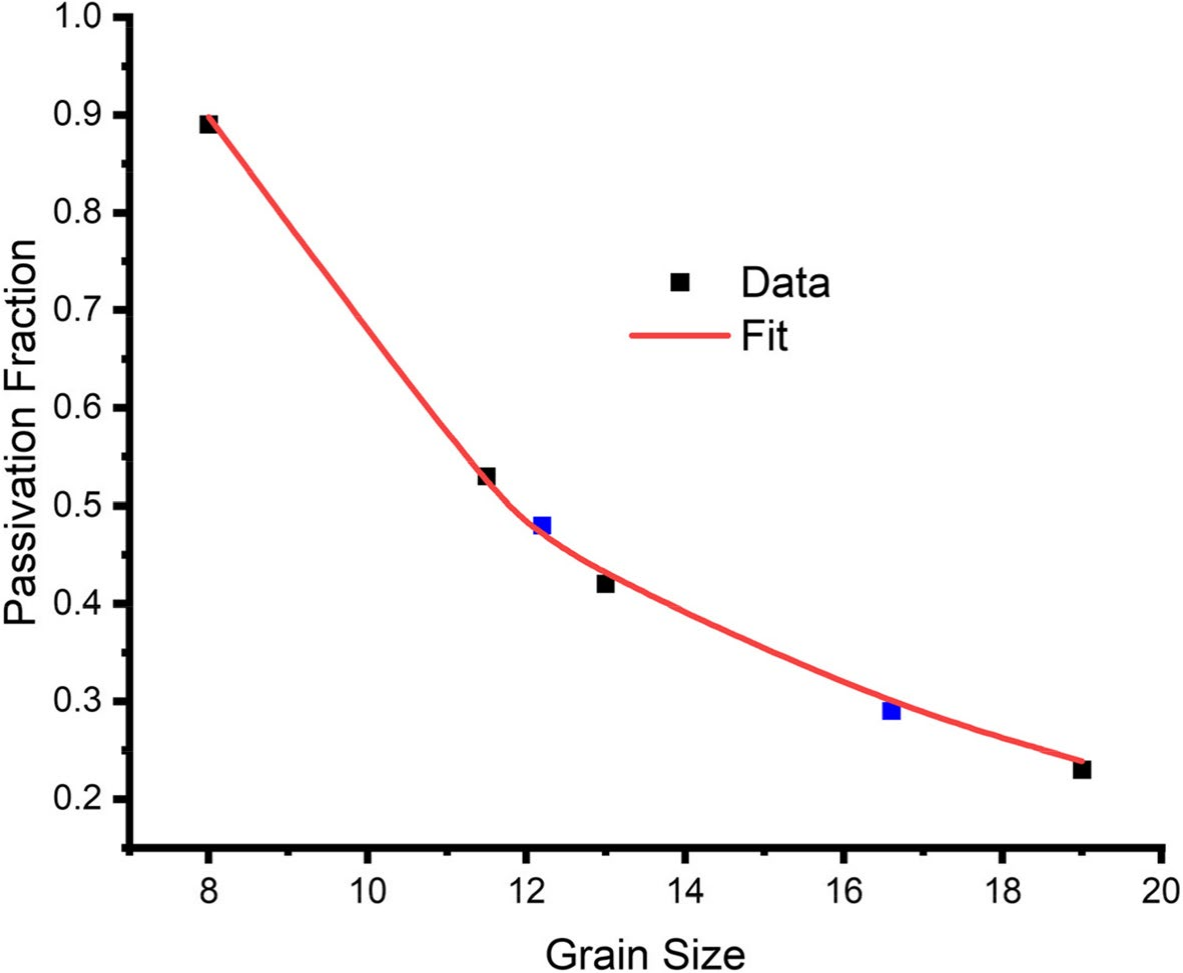
A plot of passivation fraction versus grain size showing the independent validations (blue)

**Fig. 15:**
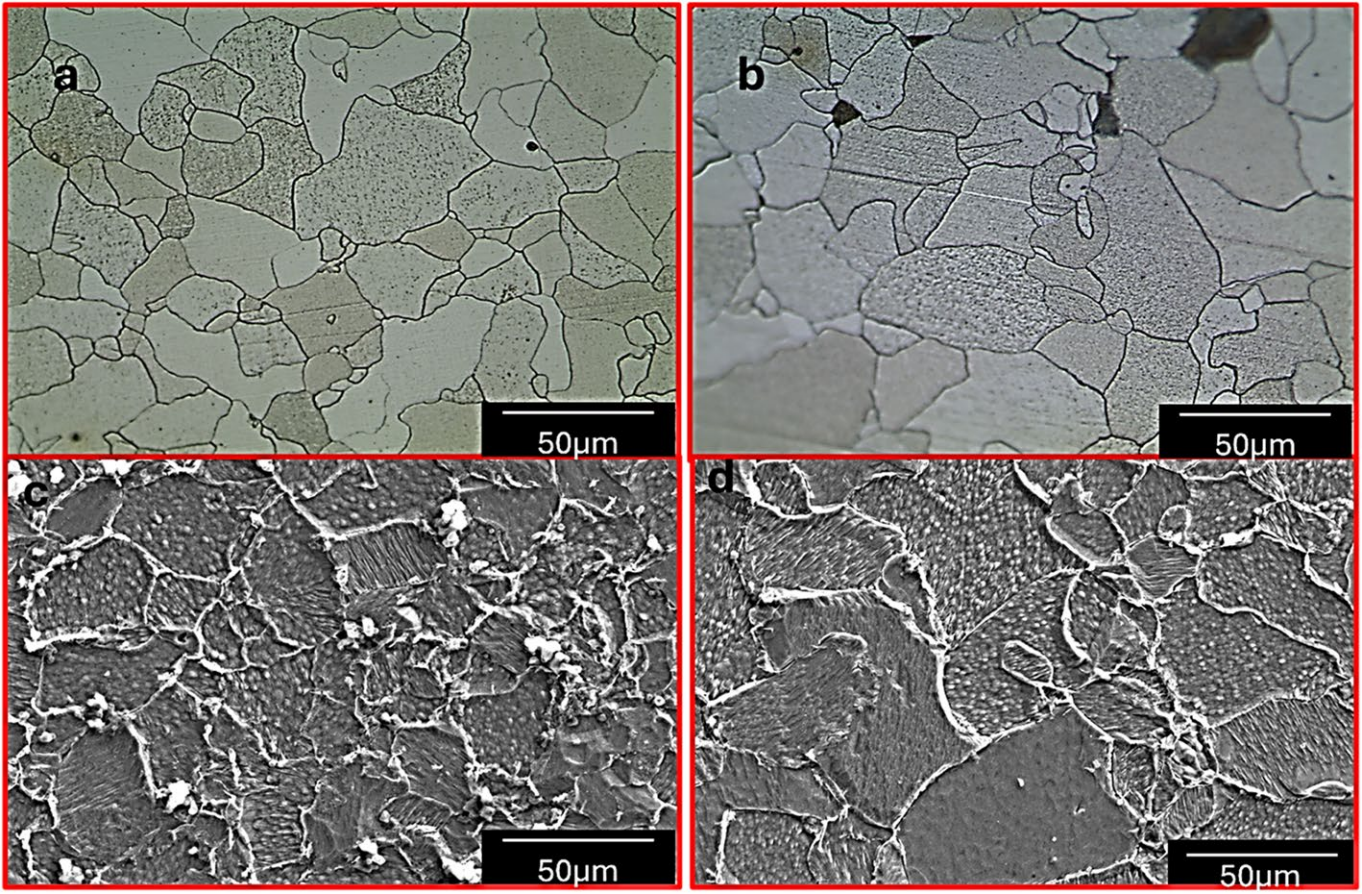
Optical micrograph and SEM image of the independent annealed samples **a**, **c** 12.2 µm, **b**, **d** 16.6 µm

In Fig. 14, two independent validations of the model, both within the bounds of the experimental data set, are represented by blue squares along the fitted line. These correspond to passivation fractions of 0.48 and 0.29, with grain sizes of 12.2 µm and 16.6 µm, respectively. The SEM image of the validation corroded samples, and the optical micrographs of the validation samples are also shown in Fig. 15. Independent validations were conducted to verify the accuracy of the phenomenological model. Optical micrographs highlighted variations in grain size, measuring 12.2 µm and 16.6 µm, corresponding to annealing temperatures of 1040 °C and 1100 °C, respectively. Additionally, the SEM images demonstrated that the passivation fraction increased as grain size decreased. Specifically, a passivation fraction of 0.48 corresponded to a grain size of 12.2 µm, while a passivation fraction of 0.29 was associated with a grain size of 16.6 µm.

Ultimately, this model demonstrates that when grain size decreases, the contribution of grain boundaries to passivation becomes more prominent since the increase in passivation with lower grain sizes is predominantly caused by higher activity at grain boundaries, and this is valid when other microstructural parameters like crystallographic texture, grain boundary character, dislocation density, and phase composition are insignificantly varied in the samples investigated.

## Conclusions

This study explored the effects of grain size, ranging from 8 to 19 µm, on the corrosion and passivation behavior of pipeline steel samples in an acidic environment. The following conclusions have been drawn from the results:

1. In acidic environments, reducing grain size significantly improves corrosion resistance. Steels with smaller grains exhibit a higher tendency to passivate, making them more resistant to corrosion.
2. Grain boundaries play an important role in corrosion protection by serving as ideal sites for oxide formation. These boundaries facilitate the rapid development of passivation layers, thereby enhancing corrosion resistance.
3. The heightened passivation observed in smaller grain sizes in an acidic environment is supported by the increased activity at grain boundaries. This makes steels with finer grains more effective at resisting corrosive environments, and this is devoid of the contribution of dislocation density when other microstructural features are unchanged.

These findings demonstrate the importance of grain size in determining the corrosion resistance of pipeline steels in acidic environments, providing useful insights for materials engineering and corrosion management.

## Data Availability

The data required to reproduce these findings are available upon request.
